# Supplementary material for: Independent evolution of tetraloop in enterovirus oriL replicative element and its putative binding partners in virus protein 3C
Source: PeerJ. 2017 Oct 6;5:e3896. doi: 10.7717/peerj.3896 (PMC5633025; doi:10.7717/peerj.3896)
Supplement: Table S1 — Tetraloops CCCG, UGUG, CAUG and UUGG that were unique for species Enterovirus A, B, C and D and were lost upon filtration, were added and are shown in blue. The gradient coloring from red to green represents abundance heat map for the genomes with different domain d sequence. [file peerj-05-3896-s025.docx]

Table S1 Relative abundance (%) of domain d apical sequences within different enterovirus species and types.

| Loop sequence | Enterovirus | | | | | | | | | | | | | | Rhinovirus | | |
| --- | --- | --- | --- | --- | --- | --- | --- | --- | --- | --- | --- | --- | --- | --- | --- | --- | --- |
|  | A | | | | B | C | | | D | E | F | G | H | J | A | B | C |
|  | all | EV71 | EV71 C4 genotype | non EV71 |  | all | PV | non PV |  |  |  |  |  |  |  |  |  |
| Triloops | | | | | | | | | | | | | | | | | |
| CCG |  |  |  |  |  | 0.4 |  | 0.8 |  |  |  |  |  |  |  |  |  |
| CAG |  |  |  |  |  | 0.4 |  | 0.8 |  |  |  |  |  |  |  |  |  |
| UCU |  |  |  |  |  |  |  |  |  |  |  |  |  |  | 0.8 | 13.5 |  |
| UUU |  |  |  |  |  |  |  |  |  |  |  |  |  |  |  | 45.9 |  |
| UAU |  |  |  |  |  |  |  |  |  |  |  |  |  |  |  | 21.6 |  |
| AUU |  |  |  |  |  |  |  |  |  |  |  |  |  |  |  | 10.8 |  |
| UGU |  |  |  |  |  |  |  |  |  |  |  |  |  |  |  | 2.7 |  |
| UUC |  |  |  |  |  |  |  |  |  |  |  |  |  |  |  | 2.7 |  |
| GAU |  |  |  |  |  |  |  |  |  |  |  |  |  |  |  | 2.7 |  |
| YNMG Tetraloops | | | | | | | | | | | | | | | | | |
| UACG | 15.0 | 9.0 |  | 22.4 | 20.9 | 38.3 | 41.0 | 34.7 |  |  |  | 37.5 | 50.0 | 40.0 | 31.9 |  | 40.5 |
| UGCG | 20.2 | 0.6 |  | 44.1 | 12.7 | 15.5 | 19.9 | 9.9 |  |  |  | 25.0 | 50.0 |  | 1.7 |  |  |
| UUCG | 2.8 | 5.1 | 6.9 |  | 1.2 |  |  |  | 86.2 |  |  |  |  |  | 5.0 |  | 16.2 |
| UCCG | 0.4 |  |  | 0.8 | 4.5 | 0.4 | 0.6 |  |  |  |  |  |  |  | 44.5 |  | 27.0 |
| CACG | 8.5 | 9.0 |  | 7.9 | 40.2 | 36.5 | 34.6 | 38.8 | 1.7 |  |  | 12.5 |  | 40.0 | 4.2 |  |  |
| CGCG | 0.5 | 0.6 |  | 0.4 | 1.2 | 4.7 | 3.8 | 5.8 |  |  |  | 12.5 |  |  |  |  |  |
| CUCG | 23.4 | 40.8 | 62.1 | 2.0 | 2.0 | 0.7 |  | 1.7 | 3.4 |  |  |  |  |  | 0.8 |  | 8.1 |
| CCCG | 7.1 | 12.5 | 13.8 | 0.4 | 6.6 | 0.4 |  | 0.8 | 1.7 |  |  |  |  |  | 10.1 |  | 5.4 |
| UAAG | 1.8 | 3.2 |  |  | 0.8 |  |  |  |  |  |  |  |  |  |  |  |  |
| UGAG | 3.9 | 7.1 |  |  | 0.4 |  |  |  |  |  |  |  |  |  |  |  |  |
| UUAG |  |  |  |  |  |  |  |  |  |  |  |  |  |  |  |  |  |
| UCAG |  |  |  |  |  |  |  |  |  |  |  |  |  |  |  |  |  |
| CAAG | 0.2 | 0.3 |  |  | 1.6 | 0.4 |  | 0.8 |  |  |  |  |  | 20.0 |  |  |  |
| CGAG | 0.2 |  |  | 0.4 |  | 0.7 |  | 1.7 |  |  |  |  |  |  |  |  |  |
| CUAG |  |  |  |  |  |  |  |  |  |  |  |  |  |  |  |  |  |
| CCAG |  |  |  |  |  |  |  |  |  |  |  |  |  |  |  |  |  |
| YACG |  |  |  |  | 0.4 |  |  |  |  |  |  |  |  |  |  |  |  |
| YNUG Tetraloops | | | | | | | | | | | | | | | | | |
| UAUG | 9.6 | 0.3 |  | 20.9 |  |  |  |  |  |  |  |  |  |  | 0.8 |  |  |
| UGUG | 0.2 |  |  | 0.4 | 0.4 | 0.4 |  | 0.8 |  |  |  |  |  |  |  |  |  |
| UUUG |  |  |  |  |  |  |  |  | 1.7 |  |  |  |  |  |  |  |  |
| UCUG |  |  |  |  | 0.4 |  |  |  |  |  |  |  |  |  |  |  |  |
| CAUG |  |  |  |  | 3.7 | 0.4 |  | 0.8 |  |  |  |  |  |  |  |  |  |
| CGUG | 0.2 |  |  | 0.4 | 1.2 | 0.7 |  | 1.7 |  |  |  |  |  |  |  |  |  |
| CUUG | 6.0 | 10.9 | 16.7 |  | 1.2 |  |  |  | 3.4 |  |  |  |  |  |  |  |  |
| CCUG | 0.2 | 0.3 | 0.5 |  | 0.4 | 0.4 |  | 0.8 |  |  |  |  |  |  |  |  |  |
| GYYA Tetraloops | | | | | | | | | | | | | | | | | |
| GCUA |  |  |  |  |  |  |  |  |  | 11.1 | 65.0 |  |  |  |  |  |  |
| GCCA |  |  |  |  |  |  |  |  |  |  | 15.0 |  |  |  |  |  |  |
| GUUA |  |  |  |  |  |  |  |  |  | 11.1 | 15.0 | 12.5 |  |  |  |  |  |
| Other tetraloops | | | | | | | | | | | | | | | | | |
| UUGG |  |  |  |  |  |  |  |  | 1.7 |  |  |  |  |  |  |  |  |
| CUUC |  |  |  |  |  |  |  |  |  |  |  |  |  |  |  |  | 2.7 |
| AUUA |  |  |  |  |  |  |  |  |  |  | 5.0 |  |  |  |  |  |  |
| Pentaloops | | | | | | | | | | | | | | | | | |
| GCUUA |  |  |  |  |  |  |  |  |  | 38.9 |  |  |  |  |  |  |  |
| GUUUA |  |  |  |  |  |  |  |  |  | 11.1 |  |  |  |  |  |  |  |
| GCCUA |  |  |  |  |  |  |  |  |  | 22.2 |  |  |  |  |  |  |  |
| GCGUA |  |  |  |  |  |  |  |  |  | 5.6 |  |  |  |  |  |  |  |
| GCGUA |  |  |  |  |  |  |  |  |  | 5.6 |  |  |  |  |  |  |  |
| GAUUA |  |  |  |  |  |  |  |  |  | 5.6 |  |  |  |  |  |  |  |
| GUCUA |  |  |  |  |  |  |  |  |  | 38.9 |  |  |  |  |  |  |  |
