# Supplementary material for: Independent evolution of tetraloop in enterovirus oriL replicative element and its putative binding partners in virus protein 3C
Source: PeerJ. 2017 Oct 6;5:e3896. doi: 10.7717/peerj.3896 (PMC5633025; doi:10.7717/peerj.3896)
Supplement: Table S2 [file peerj-05-3896-s026.docx]

Table S 2 Variety of domain d apical loop sequence in genomes of Enterovirus A species.

| N | **Loop sequence** | **Abundance** | **Abundance in filtered set of genomes** | **Diversity of 3 flanking base pairs** | **Diversity of 3 flanking base pairs in filtered set of genomes** |
| --- | --- | --- | --- | --- | --- |
| **YNMG Tetraloops** | | | | | |
| 1 | **CUCG** | 232 | 132 | 4 | 4 |
| 2 | **CCCG** | 191 | 40 | 4 | 3 |
| 3 | **UACG** | 157 | 85 | 13 | 13 |
| 4 | **UGCG** | 153 | 114 | 8 | 8 |
| 5 | **CACG** | 79 | 48 | 10 | 10 |
| 6 | **UGAG** | 45 | 22 | 2 | 2 |
| 7 | **UUCG** | 21 | 16 | 3 | 3 |
| 8 | **UAAG** | 15 | 10 | 3 | 3 |
| 9 | **CGCG** | 5 | 3 | 3 | 3 |
| 10 | **UCCG** | 2 | 2 | 1 | 1 |
| 11 | **CGAG** | 1 | 1 | 1 | 1 |
| 12 | **CAAG** | 1 | 1 | 1 | 1 |
| **YNUG Tetraloops** | | | | | |
| 13 | **UAUG** | 87 | 54 | 3 | 3 |
| 14 | **CUUG** | 57 | 34 | 2 | 2 |
| 15 | **CCUG** | 4 | 1 | 1 | 1 |
| 16 | **CGUG** | 1 | 1 | 1 | 1 |
| 17 | **UGUG** | 1 | 0 | 1 | 0 |
| **Total** | | 1052 | 564 | 61 | 59 |
