# Supplementary material for: Independent evolution of tetraloop in enterovirus oriL replicative element and its putative binding partners in virus protein 3C
Source: PeerJ. 2017 Oct 6;5:e3896. doi: 10.7717/peerj.3896 (PMC5633025; doi:10.7717/peerj.3896)
Supplement: Table S3 [file peerj-05-3896-s027.docx]

Table S 3 Variety of domain d apical loop sequence in genomes of *Enterovirus B* species.

| N | **Loop sequence** | **Abundance** | **Abundance in filtered set of genomes** | **Diversity of 3 flanking base pairs** | **Diversity of 3 flanking base pairs in filtered set of genomes** |
| --- | --- | --- | --- | --- | --- |
| **YNMG Tetraloops** | | | | | |
|  | **CACG** | 146 | 98 | 1 | 1 |
|  | **UACG** | 69 | 51 | 3 | 3 |
|  | **UGCG** | 41 | 31 | 1 | 1 |
|  | **UCCG** | 23 | 11 | 1 | 1 |
|  | **CCCG** | 18 | 16 | 2 | 2 |
|  | **CUCG** | 6 | 5 | 1 | 1 |
|  | **CAAG** | 5 | 4 | 2 | 2 |
|  | **UUCG** | 3 | 3 | 1 | 1 |
|  | **CGCG** | 3 | 3 | 2 | 2 |
|  | **UAAG** | 2 | 2 | 1 | 1 |
|  | **UGAG** | 2 | 1 | 1 | 1 |
|  | **YACG** | 1 | 1 | 1 | 1 |
| **YNUG Tetraloops** | | | | | |
|  | **CAUG** | 9 | 9 | 1 | 1 |
|  | **CUUG** | 4 | 3 | 1 | 1 |
|  | **CGUG** | 4 | 3 | 1 | 1 |
|  | **UCUG** | 1 | 1 | 1 | 1 |
|  | **CCUG** | 1 | 1 | 1 | 1 |
|  | **UGUG** | 1 | 1 | 1 | 1 |
| **Total** | | 339 | 244 | 23 | 23 |
