# Supplementary material for: Independent evolution of tetraloop in enterovirus oriL replicative element and its putative binding partners in virus protein 3C
Source: PeerJ. 2017 Oct 6;5:e3896. doi: 10.7717/peerj.3896 (PMC5633025; doi:10.7717/peerj.3896)
Supplement: Table S4 [file peerj-05-3896-s028.docx]

Table S 4 Variety of domain d apical loop sequence in genomes of *Enterovirus C* species.

| N | **Loop sequence** | **Abundance** | **Abundance in filtered set of genomes/filtered set of genomes of poliovirus** | **Diversity of 3 flanking base pairs** | **Diversity of 3 flanking base pairs in filtered set of genomes** |
| --- | --- | --- | --- | --- | --- |
| **YNMG Tetraloops** | | | | | |
| 1 | **CACG** | 254 | 101/54 | 5 | 4 |
| 2 | **UGCG** | 232 | 43/31 | 4 | 4 |
| 3 | **UACG** | 226 | 106/64 | 2 | 2 |
| 4 | **CGCG** | 21 | 13/6 | 1 | 1 |
| 5 | **CUCG** | 2 | 2 | 1 | 1 |
| 6 | **CGAG** | 2 | 2 | 1 | 1 |
| 7 | **UCCG** | 1 | 1/1 | 1 | 1 |
| 8 | **CCCG** | 1 | 0 | 1 | 0 |
| 9 | **CAAG** | 1 | 1 | 1 | 1 |
| **YNUG Tetraloops** | | | | | |
| 10 | **CGUG** | 2 | 2 | 1 | 1 |
| 11 | **CAUG** | 1 | 0 | 1 | 0 |
| **12** | **CCUG** | 1 | 1 | 1 | 1 |
| **13** | **UGUG** | 1 | 0 | 1 | 0 |
| **Triloops** | | | | | |
| 14 | **CCG** | 1 | 1 | 1 | 1 |
| 15 | **CAG** | 1 | 1 | 1 | 1 |
| **Total** | | 747 | 274 | 23 | 19 |
