# Supplementary material for: Independent evolution of tetraloop in enterovirus oriL replicative element and its putative binding partners in virus protein 3C
Source: PeerJ. 2017 Oct 6;5:e3896. doi: 10.7717/peerj.3896 (PMC5633025; doi:10.7717/peerj.3896)
Supplement: Table S5 [file peerj-05-3896-s029.docx]

Table S 5 Variety of domain d apical loop sequence in genomes of *Enterovirus D* species.

| **N** | **Loop sequence** | **Serotype** | **Abundance** | **Abundance in filtered set of genomes** | **Diversity of 3 flanking base pairs** | **Diversity of 3 flanking base pairs in filtered set of genomes** |
| --- | --- | --- | --- | --- | --- | --- |
|  | UUCG | HEV68 | 404 | 50 | 5 | 3 |
|  | UUUG | HEV68 | 4 | 1 | 1 | 1 |
|  | CUCG | HEV68 | 3 | 2 | 1 | 1 |
|  | CCCG | HEV70 | 3 | 1 | 1 | 1 |
|  | CUUG | HEV68 | 3 | 2 | 1 | 1 |
|  | CACG | HEV94 | 1 | 1 | 1 | 1 |
|  | UUGG | HEV68 | 1 | 0 | 1 | 0 |
| **Total** | | | 419 | 57 | 11 | 8 |
