# Supplementary material for: Independent evolution of tetraloop in enterovirus oriL replicative element and its putative binding partners in virus protein 3C
Source: PeerJ. 2017 Oct 6;5:e3896. doi: 10.7717/peerj.3896 (PMC5633025; doi:10.7717/peerj.3896)
Supplement: Table S6 [file peerj-05-3896-s030.docx]

Table S 6 Variety of domain d apical loop sequence in genomes of *Enterovirus E* and *F* species.

| **N** | **Loop sequence** | **Abundance** | **Abundance in filtered set of genomes** | **Diversity of 3 flanking base pairs** | **Diversity of 3 flanking base pairs in filtered set of genomes** |
| --- | --- | --- | --- | --- | --- |
| **Enterovirus E** | | | | | |
| **First oriL** | | | | | |
|  | GCUUA | 3 | 3 | 3 | 3 |
|  | GUUA | 3 | 2 | 2 | 2 |
|  | GCUA | 2 | 2 | 2 | 1 |
|  | GUUUA | 2 | 1 | 1 | 1 |
|  | GCCUA | 1 | 1 | 1 | 1 |
|  | GCGUA | 1 | 1 | 1 | 1 |
| **Total** | | **12** | **10** | **10** | **9** |
| **Second oriL** | | | | | |
|  | GCUUA | 5 | 4 | 2 | 2 |
|  | GCCUA | 4 | 3 | 3 | 2 |
|  | GAUUA | 1 | 1 | 1 | 1 |
|  | GUCUA | 1 | 1 | 1 | 1 |
|  | GUUUA | 1 | 1 | 1 | 1 |
| **Total** | | **12** | **10** | **8** | **7** |
| **Enterovirus F** | | | | | |
| **First oriL** | | | | | |
|  | GCUA | 7 | 5 | 1 | 1 |
|  | GUUA | 3 | 2 | 1 | 1 |
|  | GCCA | 2 | 2 | 1 | 1 |
|  | AUUA | 1 | 1 | 1 | 1 |
| **Total** | | **13** | **10** | **4** | **4** |
| **Second oriL** | | | | | |
|  | GCUA | 11 | 8 | 1 | 1 |
|  | GCCA | 1 | 1 | 1 | 1 |
|  | GUUA | 1 | 1 | 1 | 1 |
| **Total** | | **13** | **10** | **3** | **3** |
