# Supplementary material for: Independent evolution of tetraloop in enterovirus oriL replicative element and its putative binding partners in virus protein 3C
Source: PeerJ. 2017 Oct 6;5:e3896. doi: 10.7717/peerj.3896 (PMC5633025; doi:10.7717/peerj.3896)
Supplement: Table S7 [file peerj-05-3896-s031.docx]

Table S 7 Variety of domain d apical loop sequence in genomes of *Enterovirus G, H* and *J* species.

| **N** | **Loop and flanking base pairs sequence** | **Abundance** | **Abundance in filtered set of genomes** | **Sequence of RNA-binding tripeptide** |
| --- | --- | --- | --- | --- |
| **Enterovirus G** | | | | |
|  | uauUACGgua | 5 | 3 | STGK |
|  | uauUGCGgua | 1 | 1 | STGK |
|  | auuUGCGgau | 1 | 1 | STGK |
|  | uauCGCGgua | 1 | 1 | STGK |
|  | uauCACGgua | 1 | 1 | STGK |
|  | cacGUUAgug | 1 | 1 | SMGK |
| **Total** | | **10** | **8** | -- |
| **Enterovirus H** | | | | |
|  | uauUGCGgua | 1 | 1 | TTGK |
|  | uauUACGgua | 2 | 1 | TTGR |
| **Total** | | **3** | **2** | -- |
| **Enterovirus J** | | | | |
|  | uauUACGgua | 3 | 2 | ATGK |
|  | uauCAAGgua | 3 | 1 | ATGK |
|  | uauCACGgua | 2 | 2 | ATGK |
| **Total** | | **8** | **5** | -- |
