# Supplementary material for: Independent evolution of tetraloop in enterovirus oriL replicative element and its putative binding partners in virus protein 3C
Source: PeerJ. 2017 Oct 6;5:e3896. doi: 10.7717/peerj.3896 (PMC5633025; doi:10.7717/peerj.3896)
Supplement: Table S8 [file peerj-05-3896-s032.docx]

Table S 8 Variety of domain d apical loop sequence in genomes of *Rhinovirus A, B* and *C* species.

| **N** | **Loop sequence** | **Abundance** | **Abundance in filtered set of genomes** | **Diversity of 3 flanking base pairs** | **Diversity of 3 flanking base pairs in filtered set of genomes** |
| --- | --- | --- | --- | --- | --- |
| **Rhinovirus A** | | | | | |
|  | UCCG | 82 | 53 | 2 | 2 |
|  | UACG | 46 | 38 | 1 | 1 |
|  | CCCG | 15 | 12 | 1 | 1 |
|  | UUCG | 6 | 6 | 1 | 1 |
|  | CACG | 5 | 5 | 1 | 1 |
|  | UGCG | 3 | 2 | 1 | 1 |
|  | CUCG | 1 | 1 | 1 | 1 |
|  | UCU | 1 | 1 | 1 | 1 |
|  | **Total** | 159 | 118 | 9 | 9 |
| **Rhinovirus B** | | | | | |
|  | UUU | 18 | 17 | 2 | 2 |
|  | UAU | 17 | 8 | 2 | 2 |
|  | UCU | 7 | 5 | 2 | 2 |
|  | AUU | 4 | 4 | 2 | 2 |
|  | UGU | 1 | 1 | 1 | 1 |
|  | UUC | 2 | 1 | 1 | 1 |
|  | GAU | 1 | 1 | 1 | 1 |
|  | **Total** | 50 | 37 | 11 | 11 |
| **Rhinovirus C** | | | | | |
|  | UACG | 15 | 15 | 3 | 3 |
|  | UCCG | 10 | 10 | 3 | 3 |
|  | UUCG | 6 | 6 | 2 | 2 |
|  | CUCG | 3 | 3 | 2 | 2 |
|  | CUUC | 1 | 1 | 1 | 1 |
|  | CCCG | 3 | 2 | 1 | 1 |
| **Total** | | 38 | 37 | 12 | 12 |
