# Supplementary material for: Independent evolution of tetraloop in enterovirus oriL replicative element and its putative binding partners in virus protein 3C
Source: PeerJ. 2017 Oct 6;5:e3896. doi: 10.7717/peerj.3896 (PMC5633025; doi:10.7717/peerj.3896)
Supplement: Table S9 [file peerj-05-3896-s033.docx]

Table S 9 Variety of putative RNA-binding tripeptide of protein 3C in genomes *of Enterovirus A* species

| **N** | **Loop sequence** | **Abundance in filtered set of genomes** | **Sequence of RNA-binding tripeptide** | | | | | | | | | |
| --- | --- | --- | --- | --- | --- | --- | --- | --- | --- | --- | --- | --- |
|  |  |  | **VGK** | | **VGR** | | **TGK** | | **IGK** | | **other** | |
|  | CUCG | 132 | SVGK | 129 | -- | -- | -- | -- | SIGK | 1 | SVGE | 1 |
|  |  |  | AVGK | 1 |  |  |  |  |  |  |  |  |
|  | CCCG | 40 | SVGK | 40 | -- | -- | -- | -- | -- | -- | -- |  |
|  | UACG | 85 | SVGK | 82 | SVGR | 3 | -- | -- | -- | -- | -- |  |
|  | UGCG | 114 | SVGK | 111 | SVGR | 1 | -- | -- | SIGK | 1 | LSRK | 1 |
|  | UAUG | 54 | SVGK | 54 | -- | -- | -- | -- | -- | -- | -- | -- |
|  | CACG | 48 | SVGK | 44 | -- | -- | STGK | 2 | SIGK | 1 | -- | -- |
|  |  |  |  |  |  |  | ATGK | 1 |  |  |  |  |
|  | CUUG | 34 | SVGK | 32 | SVGR | 2 | -- | -- | -- | -- | -- | -- |
|  | UGAG | 22 | AVGK | 21 | -- | -- | TVGK | 1 | -- | -- | -- | -- |
|  | UUCG | 16 | SVGK | 15 | -- | -- | -- | -- | -- | -- | -- | -- |
|  |  |  | AVGK | 1 |  |  |  |  |  |  |  |  |
|  | UAAG | 10 | AVGK | 10 | -- | -- | -- | -- | -- | -- | -- | -- |
|  | CGCG | 3 | SVGK | 3 | -- | -- | -- | -- | -- | -- | -- | -- |
|  | CCUG | 1 | SVGK | 1 | -- | -- | -- | -- | -- | -- | -- | -- |
|  | UCCG | 2 | SVGK | 2 | -- | -- | -- | -- | -- | -- | -- | -- |
|  | CGUG | 1 | SVGK | 1 | -- | -- | -- | -- | -- | -- | -- | -- |
|  | CGAG | 1 | -- | -- | AVGR | 1 | -- | -- | -- | -- | -- | -- |
|  | CAAG | 1 | AVGK | 1 | -- | -- | -- | -- | -- | -- | -- | -- |
| **Total** | | 564 | 548 | | 7 | | 4 | | 3 | | 2 | |
