# Supplementary material for: Independent evolution of tetraloop in enterovirus oriL replicative element and its putative binding partners in virus protein 3C
Source: PeerJ. 2017 Oct 6;5:e3896. doi: 10.7717/peerj.3896 (PMC5633025; doi:10.7717/peerj.3896)
Supplement: Table S10 [file peerj-05-3896-s034.docx]

Table S 10 Variety of putative RNA-binding tripeptide of protein 3C in genomes of *Enterovirus C* species**.**

| **N** | **Loop sequence** | **Abundance in filtered set of genomes** | **Sequence of RNA-binding tripeptide** | | | | | |
| --- | --- | --- | --- | --- | --- | --- | --- | --- |
|  |  |  | **TGK** | | **IGK** | | **PGK** | |
|  | UGCG | 43 | CTGK | 38 | -- |  | APGK | 1 |
|  |  |  | ATGK | 3 |  |  |  |  |
|  |  |  | STGK | 1 |  |  |  |  |
|  | CACG | 101 | CTGK | 94 | -- | -- | -- | -- |
|  |  |  | ATGK | 6 |  |  |  |  |
|  |  |  | STGK | 1 |  |  |  |  |
|  | UACG | 106 | CTGK | 101 | CIGK | 1 | -- | -- |
|  |  |  | ATGK | 4 |  |  |  |  |
|  | UCCG | 1 | CTGK | 1 | -- | -- | -- | -- |
|  | CGCG | 13 | CTGK | 11 | -- | -- | -- | -- |
|  |  |  | STGK | 2 |  |  |  |  |
|  | CUCG | 2 | CTGK | 1 | -- | -- | -- | -- |
|  |  |  | ATGK | 1 |  |  |  |  |
|  | CGAG | 2 | CTGK | 1 | -- | -- | -- | -- |
|  |  |  | ATGK | 1 |  |  |  |  |
|  | CGUG | 2 | STGK | 2 | -- | -- | -- | -- |
|  | CCUG | 1 | STGK | 1 | -- | -- | -- | -- |
|  | CAAG | 1 | ATGK | 1 | -- | -- | -- | -- |
|  | CAG | 1 | CTGK | 1 | -- | -- | -- | -- |
|  | CCG | 1 | CTGK | 1 | -- | -- | -- | -- |
| **Total** | | 274 | 272 | | 1 | | 1 | |
