# Supplementary material for: Independent evolution of tetraloop in enterovirus oriL replicative element and its putative binding partners in virus protein 3C
Source: PeerJ. 2017 Oct 6;5:e3896. doi: 10.7717/peerj.3896 (PMC5633025; doi:10.7717/peerj.3896)
Supplement: Table S11 [file peerj-05-3896-s035.docx]

Table S 11 Variety of putative RNA-binding tripeptide and preceding residue of protein 3C in genomes of *Rhinovirus A* species.

| N | **Loop sequence** | **Abundance in filtered set of genomes** | **Sequence of RNA-binding tripeptide and preceding residue** | | | | | | | |
| --- | --- | --- | --- | --- | --- | --- | --- | --- | --- | --- |
|  |  |  | **KIGQ** | **KIGS** | **KIGN** | **KIGV** | **KIGL** | **KVGS** | **KVGQ** | **Other** |
|  | UCCG | 53 | 33 | 3 | 4 | 1 | 10 | 1 | 1 | -- |
|  | UACG | 38 | 16 | 4 | 3 |  | 6 | 7 | 2 | -- |
|  | CCCG | 12 | 8 | -- | -- | -- | 3 | -- | -- | KVGH - 1 |
|  | UUCG | 6 | 3 | 2 | 1 | -- | -- | -- | -- | -- |
|  | CACG | 5 | 2 | 1 | -- | -- | 1 | 1 | -- | -- |
|  | UGCG | 2 | 2 | -- | -- | -- | -- | -- | -- | -- |
|  | CUCG | 1 | 1 | -- | -- | -- | -- | -- | -- | -- |
|  | UCU | 1 | -- | -- | -- | -- | -- | -- | -- | TTGK -1 |
| **Total** | | 118 | 65 | 10 | 8 | 1 | 20 | 9 | 3 | 2 |
