# Supplementary material for: Independent evolution of tetraloop in enterovirus oriL replicative element and its putative binding partners in virus protein 3C
Source: PeerJ. 2017 Oct 6;5:e3896. doi: 10.7717/peerj.3896 (PMC5633025; doi:10.7717/peerj.3896)
Supplement: Table S12 [file peerj-05-3896-s036.docx]

Table S 12 Variety of putative RNA-binding tripeptide of protein 3C in genomes of *Rhinovirus C* species.

| **N** | **Loop sequence** | **Abundance in filtered set of genomes** | **Sequence of RNA-binding tripeptide** | | |
| --- | --- | --- | --- | --- | --- |
|  |  |  | **TGN** | **VGN** | **TGH** |
|  | UACG | 15 | 12 | 1 | 3 |
|  | UCCG | 10 | 4 | 2 | 3 |
|  | UUCG | 6 | 3 | 3 | -- |
|  | CUCG | 3 | 1 | -- | -- |
|  | CUUC | 1 | 1 | -- | -- |
|  | CCCG | 2 | 2 | -- | -- |
| **Total** | | 37 | 23 | 6 | 8 |
